# Association of extracellular water/total body water ratio with protein-energy wasting and mortality in patients on hemodialysis

**DOI:** 10.1038/s41598-023-41131-3

**Published:** 2023-08-31

**Authors:** Takahiro Yajima, Kumiko Yajima

**Affiliations:** 1 Department of Nephrology, Matsunami General Hospital, Hashima Gun, Gifu, 501-6062 Japan; 2 Department of Internal Medicine, Matsunami General Hospital, Hashima Gun, Gifu, 501-6062 Japan

**Keywords:** Medical research, Nephrology

## Abstract

Bioimpedance analysis-assessed extracellular water/total body water (ECW/TBW) ratio may be a marker for mortality and poor nutritional status in hemodialysis patients. In 193 maintenance hemodialysis patients, we retrospectively investigated the relationships among ECW/TBW ratio, mortality, and protein-energy wasting (PEW). Four components—body mass index, normalized protein catabolic rate, normalized serum creatinine level, and serum albumin level—constitute the simple PEW score; this score was calculated based on the positive number of items concerning malnutrition among these four components. A score ≥ 3 indicated PEW. Patients were stratified by an ECW/TBW ratio cut-off value (0.40) and by PEW versus non-PEW status. The simple PEW score, cardiothoracic ratio, and log-transformed C-reactive protein level were independently correlated with the ECW/TBW ratio. Eighty-four patients died during follow-up (median 4.3 years). After adjustments for sex, age, hemodialysis vintage, histories of cardiovascular events and diabetes, and C-reactive protein level, a higher ECW/TBW ratio and PEW were independently related to elevated risks of all-cause death. Adding the ECW/TBW ratio to a baseline risk model including PEW significantly increased C-statistics from 0.788 to 0.835. In conclusion, the ECW/TBW ratio may be an indicator of PEW and may be a predictor of death even accounting for PEW, in hemodialysis patients.

## Introduction

Patients receiving hemodialysis frequently experience malnutrition and excessive hydration, which are strongly associated with elevated mortality risks^1–3^. A recognized type of malnutrition in this patient group is protein-energy wasting (PEW), which refers to the wasting of fat and muscle due to decreased energy and/or protein consumption and chronic inflammation^1^. According to the “International Society of Renal Nutrition and Metabolism”, PEW is diagnosed on the basis of four distinct components: biochemical markers, low body fat mass, low muscle mass, and reduced intake of energy and/or protein^1^. Based on these original components, Moreau-Gaudry et al. proposed a simple PEW score including 4 nutritional indicators: serum albumin (Alb) level, serum creatinine (Cre) level adjusted by the body surface area (BSA) (Cre/BSA), body mass index (BMI), normalized protein catabolic rate (nPCR)^4^. This simple score was validated as an indicator of death in maintenance hemodialysis patients^4–6^. Volume excess is reportedly associated with hypertension and congestive heart failure and leads to cardiovascular death^7,8^.

Recently, body composition assessment using bioimpedance analysis (BIA) is used to evaluate malnutrition and fluid overload simultaneously^9,10^. We reported that the extracellular water-to-intracellular water ratio (ECW/ICW ratio), a novel marker derived from BIA, was independently associated with the geriatric nutritional risk index (GNRI), a surrogate marker of PEW^11^. In addition, the combination of the ECW/ICW ratio and GNRI improved the predictability of mortality. Conversely, the extracellular water-to-total body water ratio (ECW/TBW ratio), established as an indicator of volume excess to set dry weight^12^, is helpful to predict cardiovascular-related and all-cause mortality^13,14^. In the dialysis population, recent investigations have demonstrated an independent negative correlation between Alb and nPCR, two elements of the simple PEW score, and the ECW/TBW ratio^15,16^. Therefore, we hypothesized that ECW/TBW ratio can indicate PEW; however, relationships among ECW/TBW ratio, mortality, and PEW remain unknown.

Our study aimed to examine correlations between ECW/TBW ratio and simple PEW score in a baseline hemodialysis patient cohort. In addition, we examined relationships among the ECW/TBW ratio, PEW, and mortality in maintenance hemodialysis patients. We also investigated whether adding ECW/TBW ratio to the PEW-included risk model could predict mortality in these patients.

## Results

### Characteristics of this study patients

The present study patients’ characteristics are summarized in Table 1. We included 193 patients undergoing hemodialysis (duration of hemodialysis: median 1.2 (0.6–6.1) years, age: mean 63.4 ± 14.0 years, men: 67.4%). A history of diabetes and CVD events was presented in 43.0% and 65.3% of the patients, respectively. The cardiothoracic ratio (CTR) was 49.4 ± 5.1%. The C-reactive protein (CRP) level was 0.17 (0.06–0.44) mg/dL. The Alb level, Cre/BSA, BMI, and nPCR were 3.7 ± 0.4 g/dL, 21.9 ± 3.9 kg/m^2^, 509 ± 162 μmol/L/m^2^, and 0.77 ± 0.17 g/kg/day, respectively. In this present study, 63 patients (32.6%) were diagnosed with PEW. A significant correlation was found between ECW/TBW ratio and simple PEW score (r = 0.447, p < 0.0001). Moreover, the ECW/TBW ratio of the PEW group was higher (0.419 ± 0.074) than that the of the non-PEW group (0.343 ± 0.073) (p < 0.0001).

**Table 1:** Baseline data of the study participants.

	Overall patients (N = 193)	G1 (N = 101)	G2 (N = 28)	G3 (N = 29)	G4 (N = 35)	P-value
Men (%)	67.4	59.4	67.9	79.3	80.0	0.055
Age (years)	63.4 ± 14.0	57.7 ± 15.0	67.1 ± 11.7	69.8 ± 6.8	71.7 ± 9.3	< 0.0001
Underlying kidney disease						0.51
Diabetic kidney disease (%)	39.9	34.7	53.6	44.8	40.0	
Nephrosclerosis (%)	19.2	16.8	17.9	24.1	22.9	
Chronic glomerulonephritis (%)	33.7	40.6	25.0	27.6	25.7	
Others (%)	7.2	7.9	3.6	3.4	11.4	
Hemodialysis duration (years)	1.2 (0.6–6.1)	1.9 (0.6–7.5)	0.9 (0.6–2.8)	5.4 (0.7–9.6)	0.6 (0.5–5.4)	0.0046
Type of vascular access						0.60
Arteriovenous fistula	75.6	75.2	82.1	82.8	65.7	
Arteriovenous graft	23.3	23.8	17.9	17.2	31.4	
Catheter	1.1	1	0	0	2.9	
Alcohol (%)	21.2	18.8	32.1	20.7	20.0	0.53
Smoking (%)	23.8	21.8	32.1	31.0	17.1	0.40
Hypertension (%)	95.3	94.1	96.4	100	94.3	0.35
Diabetes mellitus (%)	43.0	36.6	50.0	51.7	48.5	0.31
Cardiovascular events (%)	65.8	55.4	82.1	75.9	74.3	0.012
BMI (kg/m^2^)	21.9 ± 3.9	22.0 ± 3.8	22.6 ± 4.4	22.2 ± 4.6	20.6 ± 2.8	0.18
BMI < 23.0, N (%)	127 (65.8)	60 (59.4)	77 (59.2)	17 (58.6)	30 (85.7)	0.019
BUN (mg/dL)	59.9 ± 16.0	65.7 ± 16.0	49.1 ± 9.5	64.3 ± 14.6	47.9 ± 9.1	< 0.0001
Creatinine (mg/dL)	9.0 ± 3.0	10.4 ± 2.8	6.1 ± 2.1	9.4 ± 1.9	7.2 ± 2.1	< 0.0001
Cre/BSA	509 ± 162	583 ± 147	341 ± 127	526 ± 101	415 ± 120	< 0.0001
Cre/BSA < 380, N (%)	44 (22.8)	4 (4.0)	21 (75.0)	1 (3.4)	18 (51.4)	< 0.0001
Alb (g/dL)	3.7 ± 0.4	3.8 ± 0.3	3.6 ± 0.4	3.7 ± 0.2	3.3 ± 0.4	< 0.0001
Alb < 3.8 g/dL, N (%)	105 (54.4)	34 (33.7)	20 (71.4)	16 (55.2)	33 (94.3)	< 0.0001
Hemoglobin (g/dL)	10.8 ± 1.4	10.9 ± 1.2	11.1 ± 1.1	10.4 ± 1.4	10.5 ± 2.0	0.20
Total cholesterol (mg/dL)	156 ± 36	157 ± 32	160 ± 38	154 ± 31	148 ± 48	0.55
Ca (mg/dL)	9.1 ± 0.8	9.1 ± 0.8	8.7 ± 0.6	9.4 ± 1.0	8.9 ± 0.7	0.015
P (mg/dL)	5.1 ± 1.4	5.5 ± 1.3	4.3 ± 0.9	5.1 ± 1.3	4.5 ± 1.3	< 0.0001
iPTH (pg/mL)	117 (51–230)	143 (63–239)	163 (73–279)	72 (25–134)	103 (24–227)	0.0084
Glucose (mg/dL)	140 ± 59	133 ± 51	143 ± 84	143 ± 51	157 ± 61	0.21
CRP (mg/dL)	0.17 (0.04–0.44)	0.12 (0.06–0.25)	0.20 (0.10–0.53)	0.21 (0.08–0.56)	0.55 (0.25–1.26)	< 0.0001
nPCR	0.77 ± 0.17	0.83 ± 0.18	0.65 ± 0.08	0.79 ± 0.16	0.65 ± 0.08	< 0.0001
nPCR < 0.80, N (%)	117 (60.6)	44 (43.6)	28 (100)	10 (34.5)	35 (100)	< 0.0001
CTR (%)	49.4 ± 5.1	48.1 ± 5.2	48.6 ± 4.3	52.5 ± 5.0	51.5 ± 3.9	< 0.0001
Simple PEW score	2.04 ± 1.06	1.41 ± 0.67	3.25 ± 0.44	1.52 ± 0.69	3.31 ± 0.47	< 0.0001
PEW, N (%)	63 (32.6)	0 (0)	28 (100)	0 (0)	35 (100)	< 0.0001
Kt/V for urea	1.3 ± 0.3	1.4 ± 0.3	1.2 ± 0.3	1.2 ± 0.3	1.3 ± 0.3	0.011
Ultrafiltration rate (mL/h/kg)	9.0 ± 3.9	9.6 ± 3.5	7.4 ± 3.7	10.3 ± 4.6	7.7 ± 4.3	0.0035
Dry weight (kg)	56.5 ± 11.9	57.1 ± 12.6	57.9 ± 12.9	57.2 ± 10.0	52.8 ± 9.8	0.25
TBW (kg)	27.2 ± 5.3	26.7 ± 5.8	27.2 ± 4.8	28.3 ± 3.3	27.8 ± 5.5	0.45
ICW (kg)	17.1 ± 3.7	18.3 ± 4.0	17.6 ± 3.2	15.6 ± 2.0	14.6 ± 2.8	< 0.0001
ECW (kg)	10.1 ± 3.2	8.4 ± 2.3	9.6 ± 2.1	12.7 ± 1.9	13.2 ± 3.3	< 0.0001
ECW/TBW ratio	0.368 ± 0.081	0.313 ± 0.049	0.352 ± 0.038	0.448 ± 0.038	0.472 ± 0.048	< 0.0001
Phase angle (°)	5.0 ± 1.5	5.8 ± 1.2	5.2 ± 1.0	3.9 ± 0.8	3.4 ± 0.9	< 0.0001

*G1* non-PEW and low ECW/TBW ratio group, *G2* PEW and low ECW/TBW ratio group, *G3* non-PEW and high ECW/TBW ratio group, *G4* PEW and high ECW/TBW ratio group, *G1–G4* groups 1–4, *BMI* body mass index, *BUN* blood urea nitrogen, *Ca* calcium, *Cre/BSA* serum creatinine level adjusted by body surface area, *Alb* serum albumin, *CRP* C-reactive protein, *nPCR* normalized protein catabolic rate, *CTR* cardiothoracic ratio, *iPTH* intact PTH, *P* phosphorous, *PEW* protein-energy wasting, *TBW* total body water, *ICW* intracellular water, *ECW* extracellular water.

### Associations between ECW/TBW ratio and baseline variables

According to the linear regression univariate analysis, the ECW/TBW ratio was positively correlated with male sex, age, history of diabetes and CVD events, log-transformed CRP (Log CRP), CTR, and simple PEW score and was negatively correlated with phosphorus level, Alb level, Cre/BSA, and nPCR. The multivariate linear regression analysis revealed independent positive associations of ECW/TBW ratio with male sex (β = 0.189), age (β = 0.215), history of diabetes (β = 0.142), Log CRP (β = 0.133), CTR (β = 0.213), and simple PEW score (β = 0.256) (Table 2).

**Table 2:** Regression analyses of the associations of the ECW/TBW ratio with baseline parameters.

Variables	Univariate		Multivariate	
	r	P-value	β	P-value
Age	0.470	< 0.0001	0.215	0.0029
Men	0.203	0.0047	0.189	0.0013
Diabetes mellitus	0.211	0.0032	0.142	0.014
History of CVD events	0.209	0.0036	0.004	0.95
CTR	0.357	< 0.0001	0.213	0.0016
Phosphorus	− 0.247	0.0005	− 0.060	0.34
Log-transformed CRP	0.344	< 0.0001	0.133	0.032
Alb	− 0.508	< 0.0001	–	–
BMI	0.031	0.66	–	–
Cre	− 0.425	< 0.0001	–	–
Cre/BSA	− 0.446	< 0.0001	–	–
nPCR	− 0.416	< 0.0001	–	–
Simple PEW Score	0.447	< 0.0001	0.256	0.0001

*CVD* cardiovascular disease, *CTR* cardiothoracic ratio, *CRP* C-reactive protein, *Alb* serum albumin, *BMI* body mass index, *Cre* serum creatinine, *Cre/BSA* serum creatinine level adjusted by body surface area, *nPCR* normalized protein catabolic rate, *TBW* total body water, *ECW* extracellular water, *PEW* protein-energy wasting.

### Relationships of the ECW/TBW ratio and PEW with mortality

**Figure 1:**
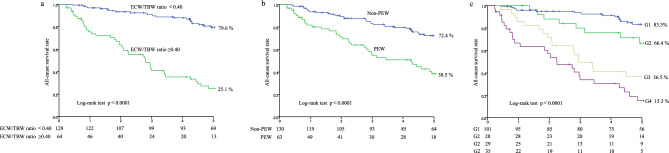
Kaplan–Meier analyses for all-cause mortality. The survival rates for the (**a**) two groups with a lower ECW/TBW ratio (ECW/TBW ratio ≤ 0.40) and a higher ECW/TBW ratio (ECW/TBW ratio > 0.40); (**b**) two groups with PEW and without PEW; and (**c**) four groups with a lower ECW/TBW ratio and no PEW (group 1: G1), a lower ECW/TBW ratio and PEW (group 2: G2), a higher ECW/TBW ratio and no PEW (group 3: G3), and a higher ECW/TBW ratio and PEW (group 4: G4). *PEW* protein-energy wasting, *ECW* extracellular water, *TBW* total body water.

Overall, 84 patients died (CVD, 46 [54.7%]; infectious disease, 22 [26.2%]; malignancy, 11 [13.1%]; and other causes, 5 [6.0%]) during a median follow-up period of 4.3 [1.9–6.8] years. The five-year survival rates in the higher and lower ECW/TBW ratio groups were 25.1% and 79.6%, respectively (p < 0.0001) (Fig. 1a), and those in the PEW and non-PEW groups were 38.5% and 72.4%, respectively (p < 0.0001) (Fig. 1b). When patients were stratified according to the cut-off value of the ECW/TBW ratio and PEW or non-PEW status, the five-year survival rates in G1, G2, G3, and G4 were 83.3%, 66.4%, 36.5%, and 15.3%, respectively (p < 0.0001) (Fig. 1c).

The univariate Cox analysis showed that a higher ECW/TBW ratio and PEW were significantly associated with elevated risks of all-cause death (HR: 5.48, 95% CI 3.49–8.59, p < 0.0001; HR: 2.77, 95% CI 1.78–4.31, p < 0.0001) (Table 3). A higher ECW/TBW ratio and PEW were independently associated with elevated risks of all-cause death even after adjustment for sex, age, hemodialysis durations, history of diabetes and CVD events, and CRP (adjusted HR [aHR]: 3.63, 95% CI 2.18–6.06, p < 0.0001; aHR: 1.79, 95% CI 1.10–2.90, p = 0.018) (Table 3). Moreover, the aHRs (vs. G1) for G2, G3, and G4 were 1.76 (95% CI 0.84–3.68, p = 0.13), 3.69 (95% CI 1.92–7.09, p < 0.0001), and 5.19 (95% CI 2.61–10.33, p < 0.0001), respectively (Table 3). In addition, the aHR for G4 vs. G2 was 2.94 (95% CI 1.40–6.20, p = 0.0045).

**Table 3:** The Cox analysis of the ECW/TBW ratio and PEW with all-cause mortality.

Variables	Univariate		Multivariate	
	Hazard ratio (95% confidence interval)	P-value	Hazard ratio (95% confidence interval)	P-value
ECW/TBW > 0.40	5.48 (3.49–8.59)	< 0.0001	3.63 (2.18–6.06)*	< 0.0001
PEW	2.77 (1.78–4.31)	< 0.0001	1.79 (1.10–2.90)*	0.018
Cross-classified (versus G1)				< 0.0001
G2	2.31 (1.15–4.64)	0.018	1.76 (0.84–3.68)*	0.13
G3	5.32 (2.94–9.62)	< 0.0001	3.69 (1.92–7.09)*	< 0.0001
G4	9.28 (5.14–16.73)	< 0.0001	5.19 (2.61–10.33)*	< 0.0001

*ECW* extracellular water, *TBW* total body water, *PEW* protein-energy wasting.

*Adjustments for age, sex, hemodialysis vintage, history of diabetes and cardiovascular disease, and C-reactive protein.

### Model discrimination

The C-index predicting all-cause death improved significantly from 0.788 to 0.835 (p = 0.038) when adding ECW/TBW ratio to the baseline risk model, which included age, sex, hemodialysis vintage, history of diabetes and CVD events, CRP, and PEW (Table 4).

**Table 4:** The predictability of the ECW/TBW ratio with all-cause mortality.

Variables	C-statistics	P-value	NRI	P-value
Baseline risk model*	0.788 (0.724–0.853)	Ref.	Ref.	
+ ECW/TBW ratio	0.835 (0.779–0.891)	0.038	0.710	< 0.0001

*NRI* net reclassification improvement, *ECW* extracellular water, *TBW* total body water, *CVD* cardiovascular disease.

*Included age, sex, hemodialysis vintage, history of diabetes and CVD, C-reactive protein, and simple protein-energy wasting score.

## Discussion

In patients receiving hemodialysis, the ECW/TBW ratio was independently correlated with simple PEW score, Log-transformed CRP, and CTR. In this population, those with greater ECW/TBW ratios and those who had PEW had higher likelihoods of dying from any cause. Those who had PEW and an elevated ECW/TBW ratio had the highest probability of dying from any cause. Additionally, the prediction of mortality was much improved when adding the ECW/TBW ratio to the simple PEW score-included risk model. The ECW/TBW ratio—a marker of PEW, inflammation, and fluid overload—may therefore be a strong predictor of mortality.

PEW, a wasting of muscle and fat, is common and highly related to mortality in patients undergoing hemodialysis. We have recently proposed that the ECW/ICW ratio, a novel but minor BIA-measured parameter, may be a marker of PEW with the ECW/ICW ratio being independently associated with the GNRI^11^. In addition, the repeated measurements of the ECW/ICW ratio and the GNRI increased the predictability for mortality^17,18^. Moreover, the combination of the ECW/ICW ratio and the GNRI improved the predictive accuracy of mortality^11^. The current study evaluated the association between the ECW/TBW ratio, one of the major established BIA-measured parameters, and PEW. PEW was diagnosed using established diagnostic criteria and not a surrogate marker. The usefulness of the WCW/TBW ratio for predicting all-cause mortality with adjustment for PEW was also investigated.

In the present study, the ECW/TBW ratio had independent and positive associations with the simple PEW score, log-transformed CRP, and CTR in patients on hemodialysis. To our knowledge, this study is the first to directly examine the association between the ECW/TBW ratio and PEW. In this present study, the ECW/TBW ratio had a significant negative correlation with each item of the simple PEW score except for BMI: Alb, r = − 0.508; Cre/BSA, r = − 0.446; nPCR, r = − 0.416; and BMI, r = 0.031 (p = 0.66). According to the original PEW criteria^1^, body mass is assessed using the BMI, body fat percentage, and unintentional weight loss. As ECW/TBW ratio had a significant association with Cre/BSA but not BMI, this might be an indicator of muscle wasting and not simply an indicator of the obesity paradox^19,20^. Furthermore, in this case, the simple PEW score, CRP, and CTR can reflect malnutrition, inflammation, and fluid volume, respectively, and these factors may interact with each other. The possible pathophysiology may be that fluid overload can introduce bowel edema; thereafter, the translocation of bowel endotoxin into the circulation may induce inflammation^21^. The inflammation can lead to malnutritional status owing to protein catabolism^22,23^, and then hypoalbuminemia may exacerbate the systemic edema. Thus, ECW/TBW ratio may indicate PEW, inflammation, and fluid overload. However, the present study was performed in a cross-sectional design; therefore, establishing causal relationships among variables is difficult. A longitudinal study is needed to confirm these findings in the future.

In this study, PEW was evaluated using a simple PEW score, and the ARNOS prospective study validated its utility for predicting mortality^4^. The study divided patients into four groups as follows: (G1) severe wasting, simple PEW score of 0 and 1; (G2) moderate wasting, simple PEW score of 2; (G3) slight wasting, simple PEW score of 3; and (G4) normal nutritional status, simple PEW score of 4. Compared with G1, G3 and G4 (simple PEW score ≥ 3) had an independent correlation with an increased mortality risk. Additionally, the original diagnostic criteria for PEW required at least three of the four components for a diagnosis of PEW. Thus, in this study, patients with a simple PEW score ≥ 3 were diagnosed as having PEW. In the present study, PEW was independently associated with an elevated all-cause death risk. Moreover, patients with a higher ECW/TBW ratio and PEW (G4) were at the highest risk of death; therefore, the combination of the ECW/TBW ratio and PEW may be useful to stratify the risks of death. Interestingly, compared with G1 and G2, G3 and G4 were associated with elevated mortality risks. The predictability of all-cause death significantly improved after the ECW/TBW ratio was added to the simple PEW score-contained baseline risk model. Therefore, the ECW/TBW ratio may be a powerful predictor of all-cause mortality in hemodialysis patients with PEW and without PEW. This might be because ECW/TBW ratio is not merely an indicator of PEW but also an indicator of inflammation and volume overload.

There were some limitations to consider. First, we included only Japanese patients receiving hemodialysis; therefore, our results may not apply to hemodialysis patients of other ethnic groups. Second, this study was a retrospective single-center study with a relatively small number of hemodialysis patients. Third, the ECW/TBW ratio was measured during study enrollment; therefore, the changes in the ECW/TBW ratio during the follow-up periods were not evaluated. To validate our study findings, multicenter prospective studies with large sample sizes and other ethnic groups may be needed in the future.

In conclusion, for hemodialysis patients, the simple PEW score, CRP, and CTR were independently correlated with the ECW/TBW ratio. An elevated ECW/TBW ratio was linked to greater risks of all-cause death. Moreover, adding the ECW/TBW ratio to the simple PEW score–containing baseline risk model significantly improved the prediction of death. The ECW/TBW ratio may not only be useful to predict PEW but also to predict inflammation and volume overload and, therefore, may be a powerful predictor of mortality.

## Methods

### Participants included in this study

Patients who had stably undergone hemodialysis (4 h/session, 3 times a week) for more than 6 months were retrospectively included. At the Matsunami General Hospital, body composition was measured using BIA between January 2008 and December 2015. Because the current investigation was carried out retrospectively by evaluating data gathered from routine clinical practice, the necessity to obtain prior informed consent was waived by the “Ethics Committee of Matsunami General Hospital”. “Ethics Committee of Matsunami General Hospital” approved the study plan, which was performed adhering to the principles of the Declaration of Helsinki (Approval No. 541).

### Data collection

The baseline information of each participant obtained from medical records was: sex; age; kidney disease that led to end-stage kidney disease, such as diabetic nephropathy, nephrosclerosis, chronic glomerulonephritis, and others; hemodialysis duration; smoking and alcohol habits; and histories of diabetes, hypertension, and cardiovascular disease (CVD) events. Diabetes was defined based on having received a previous diagnosis of diabetic retinopathy and/or a history of using glucose-lowering medication. Hypertension was defined by two parameters: (1) using some anti-hypertensive drugs and/or (2) a pre-hemodialysis blood pressure ≥ 140/90 mmHg. The CVD events included peripheral artery disease, myocardial infarction, heart failure, angina pectoris, and stroke (hemorrhage or infarction). At the start of the week, blood tests were taken in the supine position before and after a hemodialysis session. BIA was performed in the supine position about 30 min after the hemodialysis session, and ICW, ECW, and TBW values were obtained by a body composition analyzer with multiple frequencies (2.5–300 kHz) using the ankle and wrist method (MLT-550N; Sekisui Medical, Siga, Japan). The measurement was performed on Wednesday or Thursday. The CTR was calculated based on chest radiography which was performed on the same day. BMI was calculated from dry weight and height: BMI = dry weight/height^2^ (kg/m^2^).

### Definition and calculation of the simple PEW score

The simple PEW score was defined as^4^ the positive number of items regarding malnutrition among the four components (i.e., Alb, Cre/BSA, BMI, and nPCR). The cut-off values of Alb, Cre/BSA, BMI, and nPCR were defined as 3.8 g/dL, 380 μmol/L/m^2^, 23 kg/m^2^, and 0.8 g/kg/day, respectively. In this study, accounting for the original version of PEW^1,24^, PEW was diagnosed when at least three of the four components were applied.

### Grouping and study outcomes

ECW/TBW ratios were used to categorize patients into two groups: those with ratios higher than 0.40 and those with ratios lower than 0.40. The cut-off value was used as previously reported by Kim et al.^12^. Moreover, they were also separated into those with PEW and those without PEW (non-PEW). They were further cross-classified into four groups as per the presence or absence of PEW and the cut-off point of the ECW/TBW ratio: non-PEW and lower ECW/TBW ratio (Group 1: G1), PEW and lower ECW/TBW ratio (Group 2: G2), non-PEW and higher ECW/TBW ratio (Group 3: G3), and PEW and higher ECW/TBW ratio (Group 4: G4).

Patients who were alive by December 2020 were censored, and the endpoint of the present study was all-cause mortality.

### Statistical analysis

A non-normally distributed variable was reported as a median and interquartile range, while a normally distributed variable was reported as a mean and standard deviation. For categorical variables, the chi-square test was used to compare differences between groups (G1–G4) that were divided by the presence or absence of PEW and the cutoff value of the ECW/TBW ratio at 0.40. For continuous variables, the Kruskal–Wallis test or one-way analysis of variance was used to compare differences. We examined the correlation between simple PEW score and the ECW/TBW ratio using Pearson's correlation coefficient. To examine the differences in the ECW/TBW ratio between the PEW and non-PEW groups, we employed Student's t-test. We used a univariate regression analysis to evaluate at how the ECW/TBW ratio and baseline factors related. Subsequently, using factors that were substantially linked with the ECW/TBW ratio in the univariate regression analysis, we conducted a multivariate regression analysis. We did not include Alb, Cre/BSA, or nPCR in the multivariate linear regression model because they were part of the simple PEW score. We utilized the Kaplan–Meier method to estimate the survival rate and the log-rank test to examine differences in the survival rates. To calculate hazard ratios (HRs) and 95% confidence intervals (CIs) for mortality, we used a univariate Cox analysis. We performed a multivariate Cox analysis with adjustments for sex, age, and covariates, which were significant based on the univariate Cox analysis. Because Alb, Cre, BMI, and nPCR were included in the simple PEW score, these covariates were excluded in the multivariate Cox analysis. We compared the predictabilities for mortality between the baseline risk model, including PEW, and the enriched model with the ECW/TBW ratio, and we calculated the C-index and the net reclassification improvement (NRI).

We performed the statistical analyses using SPSS version 28 (IBM Corp., Armonk, NY, USA). Differences with a p-value < 0.05 were considered statistically significant.

## Data Availability

The dataset used for the present study is available from the corresponding author upon reasonable request.
